# Simple and reusable picoinjector for liquid delivery via nanofluidics approach

**DOI:** 10.1186/1556-276X-9-147

**Published:** 2014-03-25

**Authors:** Shunbo Li, Wenbin Cao, Yu Sanna Hui, Weijia Wen

**Affiliations:** 1 Department of Physics and Joint KAUST-HKUST Micro/Nano-Fluidics Laboratory, The Hong Kong University of Science and Technology, Clear Water Bay, Kowloon, Hong Kong; 2 Nano Science and Technology Program and Department of Physics, The Hong Kong University of Science and Technology, Clear Water Bay, Kowloon, Hong Kong

**Keywords:** Picoinjector, Nanofluidics, Electroosmosis, Reusable device

## Abstract

Precise control of sample volume is one of the most important functions in lab-on-a-chip (LOC) systems, especially for chemical and biological reactions. The common approach used for liquid delivery involves the employment of capillaries and microstructures for generating a droplet which has a volume in the nanoliter or picoliter range. Here, we report a novel approach for constructing a picoinjector which is based on well-controlled electroosmotic (EO) flow to electrokinetically drive sample solutions. This picoinjector comprises an array of interconnected nanochannels for liquid delivery. Such technique for liquid delivery has the advantages of well-controlled sample volume and reusable nanofluidic chip, and it was reported for the first time. In the study of the pumping process for this picoinjector, the EO flow rate was determined by the intensity of the fluorescent probe. The influence of ion concentration in electrolyte solutions over the EO flow rate was also investigated and discussed. The application of this EO-driven picoinjector for chemical reactions was demonstrated by the reaction between Fluo-4 and calcium chloride with the reaction cycle controlled by the applied square waves of different duty cycles. The precision of our device can reach down to picoliter per second, which is much smaller than that of most existing technologies. This new approach, thus, opens further possibilities of adopting nanofluidics for well-controlled chemical reactions with particular applications in nanoparticle synthesis, bimolecular synthesis, drug delivery, and diagnostic testing.

**PACS:**

85.85.+ j; 87.15.hj; 82.39.Wj

## Background

Precise control of the sample volume is the first prerequisite in high-resolution micro total analysis systems (μTAS) and microreactors [1-3]. Nanopipettes [4] and picoinjectors [5] are major ways to achieve this aim. However, the existing techniques utilizing either carbon nanotubes or electromicrofluidics are cumbersome to fabricate and difficult to operate. Chen et al. [6] developed a nanoinjector based on atomic force microscopy (AFM). This technique is limited by the throughput and difficulty in control of liquid volume. Seger et al. [7] demonstrated single-cell surgery by a nanopipette. It is applied to penetrate the cell membrane by mechanical force. Sometimes, one has to adjust the surrounding medium outside of cells for biochemical reactions. The embedded pumps are regarded as portable and stand-alone systems for this application. Yokokawa et al. [8] invented an on-chip syringe pump for picoliter liquid manipulation by integrating sliders of an electrostatically controlled linear inchworm actuator made by a piezoelectric material. However, the drawback of the on-chip syringe pump is the complex fabrication method involving a multistructured MEMS procedure. Unlike traditional micropipette injection and on-chip syringe pump methods which rely on pressure differences, we proposed direct delivery of liquid using an electrical signal in μTAS. This is another novel approach for constructing a picoinjector with high precision and without mechanical movements. This technique is based on the fact that fluid and nanoparticles have interesting properties in nanoscaled pores or channels [9,10]. It is due to the large effect of the electrical double layer which is comparable to the pore or channel size. Electrokinetic phenomena [11,12] generated by an electric field in microscopic scale are most promising to deliver pure water, pure polar organic solvents, inorganic buffer, and bio-macromolecules in nanochannels. Electroosmotic pumps [13], based on electrokinetics and operated with no moving part, are a better way for liquid delivery since they are much easier to integrate in μTAS than the piezoelectric method. They are driven by electroosmosis (EO) which arises from the existence of an electrical double layer at the solid-liquid interface and holds great promise in generating fluid flow in nanochannels under the influence of an electric field. Transport of analytes in nanochannels has been well studied by Pennathur and Santiago [14], and the concept can be conveniently adopted in our picoinjector. The electroosmosis-based picoinjector possesses an array of one-dimensional (1D) nanochannels for precise fluid transfer under the condition of applying the controlling signal. Potential applications based on this picoinjector include precisely controlled chemical reactions [15], drug delivery [16], as well as biomolecular translocation [17]. All of these applications are based on the variation of the applied voltage bias across nanopores or nanochannels.

In this paper, we reported a new approach of a picoinjector by means of 1D nanochannels which offers precise control of solution volume on the scale of picoliter. The injection rate or pumping rate was determined by measuring the fluorescent intensity subsequent to the injection of the fluorescent solution into the connected microchannel. Solutions of different ion concentrations were also utilized for simulating various scenarios. Moreover, microreaction between Fluo-4 and calcium ions was successfully demonstrated by our picoinjector to show the capability of our device in terms of its controllability of chemical reaction in a continuous phase.

### Physics background

**Figure 1:**
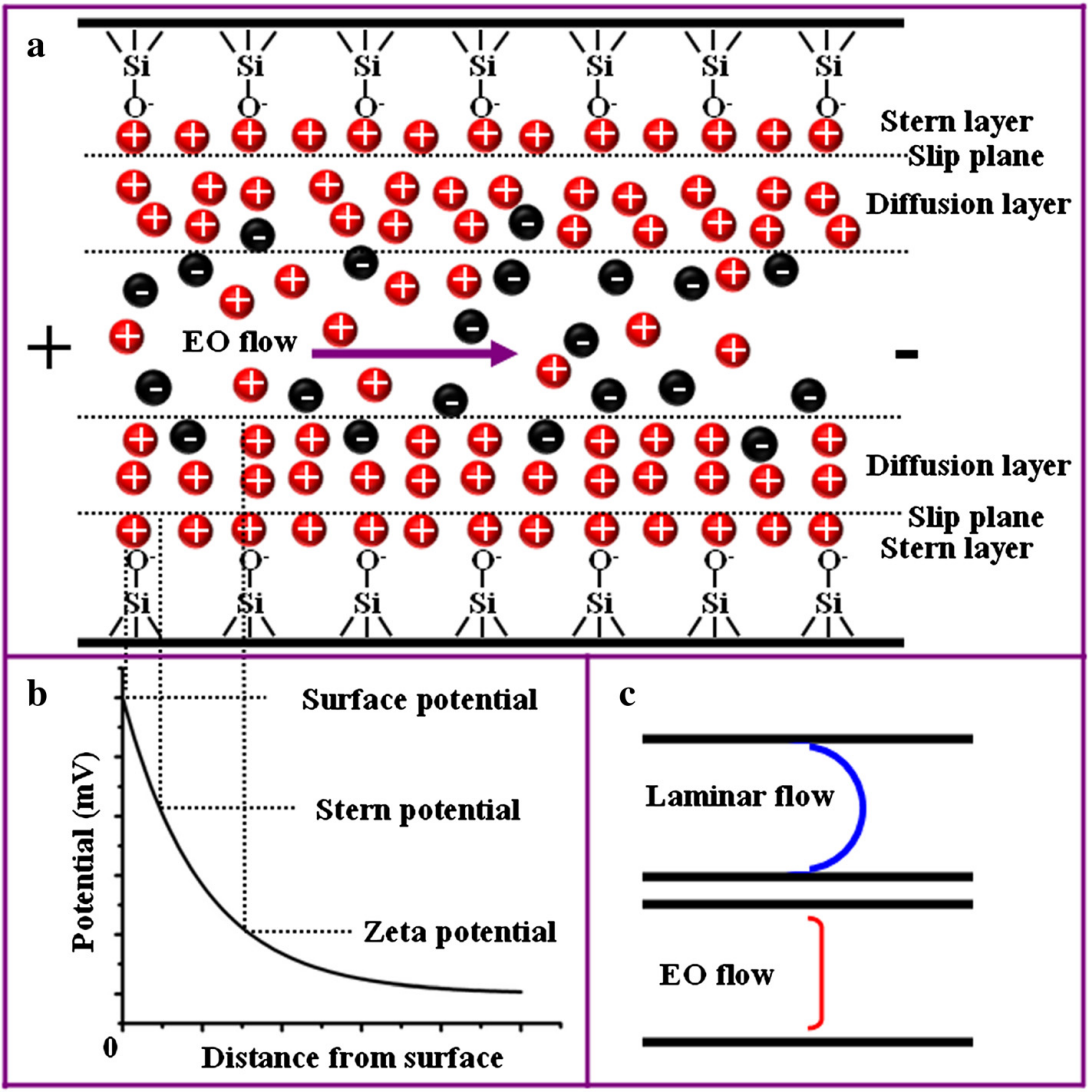
**Depiction of the interior of a silica nanochannel in the presence of a buffer solution. (a)** Schematic showing the EDL and EO flow. **(b)** The corresponding potential at different layers. **(c)** Flow profiles of the laminar and electroosmotic flows when the channel dimension is beyond the electric double layer overlapping regime.

The origin of electroosmotic flow (EOF) is directly related to the electrical double layer (EDL) which comes from the ionization of silanol (SiOH) groups when the silica channel is filled with a buffer solution. Such reaction is represented by SiOH  ⇌ SiO^-^  +  H^+^. The silanol groups on the surface are ionized, forming a wall of negatively charged silanoate (SiO^-^) groups that are catalyzed by the OH^-^ ions in the solution. The positive counterions compensate the wall of negative charge so that EDL is formed near the silica wall. The schematic illustration of this phenomenon is shown in Figure 1. The Stern layer is closest to the surface at which the positive charges are tightly held by the solid-liquid interface, while the next layer is the diffusion layer as depicted respectively in Figure 1a. The predominance of the positive ions in the diffusive region can be accounted by a negative potential, *ζ* potential, which serves as the boundary condition for the so-called Debye layer. The surface potential, Stern potential, and zeta potential and their respective locations within the nanochannel are illustrated in Figure 1b. When the external electric field is applied along the channel, the positive counterions that are attracted to the negatively charged wall move toward the cathode, resulting in electroosmotic flow. Figure 1c compares the velocity profile of the laminar flow and the electroosmotic flow across the channel width. Laminar flow is generated by the pressure difference within the channel; thus, the flow profile is greatly influenced by the interaction between the flowing liquid and the channel wall. The small fluidic velocity near the channel wall is the result of a large drag force between the silica channel wall and the water solution. On the other hand, EOF is induced by the mobility of charges near the channel wall. Hence, the flow velocity is almost the same in a certain range of the channel size. It is noted that EOF has a limited effect when the channel size is larger than 1 μm due to the fact that EDL is usually very thin (in the order of nanometers). The velocity of EOF is given by the Smoluchowski equation:

(1) $${\vec{u}}_{\text{EO}}=\varepsilon_{0}\varepsilon_{r}\zeta \vec{E}/\eta$$

where *ε*_0_ is the permittivity of vacuum, *ε*_*r*_ is the relative permittivity of the filled solution, *ζ* is the zeta potential of EDL, *E* is the applied electric field, and *η* is the dynamic viscosity of the solution.

The zeta potential can be quantified by the well-known Poisson equation for an arbitrary-shaped charged surface:

(2) $$\nabla^{2}\psi =-\rho /\left(\varepsilon_{0}\varepsilon_{r}\right)$$

where ∇^2^ is the Laplacian operator, *ψ* is the potential at a given position within the EDL, and *ρ* is the charge density. This equation can be further simplified using the Debye-Hückel approximation [18]:

(3) $$\nabla^{2}\psi =k^{2}\psi$$

where 1/*k* is the Debye length.

It is concluded that the ion concentration in the filled solution will affect the EOF velocity by altering the zeta potential of EDL as suggested by Equations 1 and 2. A higher ion concentration of the solution results in lower EOF velocity due to the larger capability to balance the negative charges at the channel wall, and thus, the EDL will be narrowed. This character of variation of EDL can also be expressed by the Debye length which is closely related to the zeta potential as seen in Equation 3. A larger Debye length means a higher zeta potential of EDL and larger EOF velocity. It was reported that the Debye length of silica filled with a 10 μM monovalent ion solution was 100 nm, compared to 0.3 nm when silica was immersed in a 1 M monovalent ion solution [19].

## Methods

### Chip fabrication

A two-step deep reactive ion etching (DRIE) was performed to achieve a microreactor chip containing a picoinjector based on a 1D nanochannel. The first step of DRIE was conducted to fabricate the 1D nanochannel junction for liquid delivery. Then, the second step of DRIE was conducted to form microchannels for the chemical reaction. Conventional photolithography and photoresist stripping processes were employed to construct channels with the desired depth. A silicon (Si) wafer was cleaned in H_2_SO_4_:H_2_O_2_ solution (volume ratio of 10:1) at 120°C for 10 min, followed by deionized water (DI) for 4 cycles, then HF:H_2_O solution (1:50) at 22°C for 1 min and DI water for 4 cycles, and finally spin-dried in hot N_2_ gas for 15 min. Then, the Si wafer was processed by hexamethyldisiloxane (HMDS) coating and positive photoresist HPR 504 spin-coated at 4,000 rpm for 30 s. The wafer was soft-baked on a hot plate at 110°C for 60 s before exposing to UV via the Mask Aligner (SUSS Microtec MA6-2, Garching Germany) for 5 s. The photoresist was developed using FHD-5 for 60 s and post-baked on a hot plate at 120°C for 60 s. The micropatterns were successfully defined at this stage. The Si wafer was then etched by a DRIE machine (Surface Technology Systems, Newport, UK) and followed by photoresist stripping in PS210 Photoresist Asher (PVA Tepla AG, Kirchheim, Germany) for 25 min. After constructing the microchannels, 10 nm of thermal oxide was grown using a diffusion furnace to form silica on the channel wall.

After drilling the inlets and outlets on the Si chip by a mechanical driller, the chip has to be sealed to form a closed channel. A thin film of polydimethylsiloxane (PDMS) was applied for such purpose due to the good adhesion between PDMS and the Si chip. PDMS was formulated from Sylgard 184 silicone elastomer mixture (Dow Corning Corporation, Midland, MI, USA) at a weight ratio of base:curing agent = 10:1. Then, it was poured onto a Si wafer with saline coating on the surface and pressed against a cleaned glass slide. After curing PDMS in an oven at 60°C for 2 h, the microchip was constructed by pressing the Si chip against the glass slide with the thin layer of PDMS on its surface.

**Figure 2:**
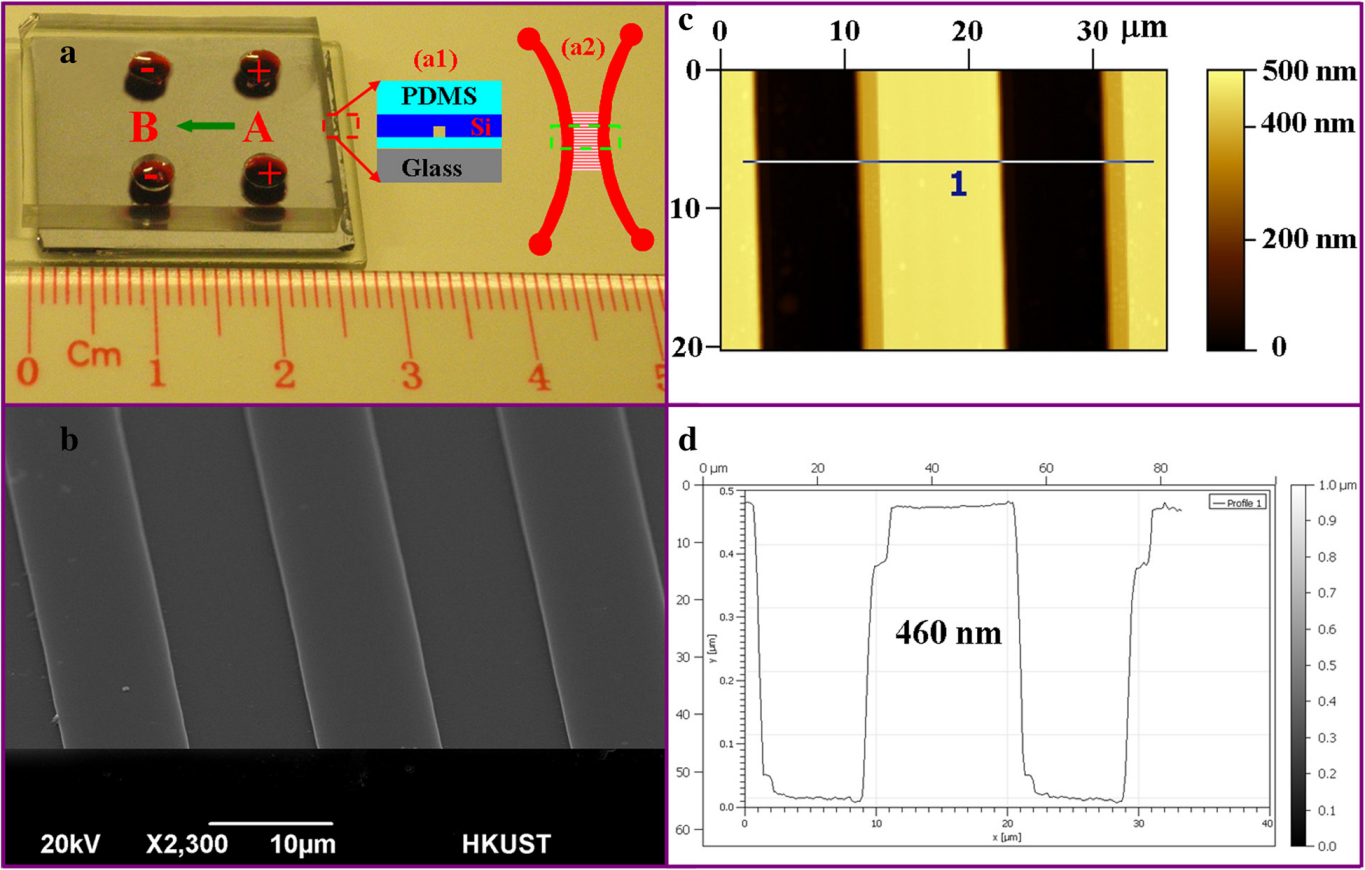
**Fabricated chip with a picoinjector. (a)** The optical image of the device showing the multilayer structures. The insets show the schematic illustrations of the fabricated layers (a1) and the channel configuration (a2) which consists of two main microchannels and interconnected by the nanochannel array (20 channels). **(b)** The SEM image of the nanochannel array with a channel width of 10 μm. **(c)** The AFM image showing the topological profile of the nanochannel array. **(d)** The depth profile along the line in **(c)** confirming that the depth of a single nanochannel is 460 nm.

The fabricated microchip is shown in Figure 2a. The microreactor is comprised of two microchannels: channels A and B with a width of 300 μm and a depth of 12 μm and an array (20 channels) of 1D nanochannels that connected the two microchannels to demonstrate the injection process. It is not necessary to adopt 20 nanochannels. One can increase or decrease the number according to their applications. Fewer nanochannels will result in higher precision, and more nanochannels will give a higher throughput. The inset (a1) in Figure 2a illustrates the multilayer structure showing the PDMS, the silicon chip, and the glass slide. Another inset (a2) shows the structure of the two microchannels connected by the nanochannel array that is highlighted by the green dashed square. When the electric field across channel A and channel B was applied, fluid flowed from channel A to channel B through the nanochannel array as indicated by the green arrow in the same figure. The enlarged scanning electron microscopy (SEM) image of the nanochannel array is shown in Figure 2b. The channel width observed was 10 μm. The more accurate measurement of the nanochannel depth was conducted by atomic force microscopy (AFM) with the results illustrated in Figure 2c. The depth of the nanochannel was determined to be 460 nm as shown in Figure 2d with respect to the line profile defined in Figure 2c.

### Materials and methods

A fluorescent dye solution was used in our experiment for the determination of the pumping rate from one microchannel to another. A pH 7.0 phosphate buffer solution (PBS) with a K_2_HPO_4_ concentration of 27.5 mM and a KH_2_PO_4_ concentration of 20.0 mM was prepared as the standard solution since many biochemical reactions are conducted in this buffer solution. Then, analyte solutions with specific ion concentrations were prepared by diluting the standard PBS. The dilution of the standard PBS is denoted by ‘a × PBS,’ where ‘1/a’ denotes the dilution factor, e.g., ‘0.1× PBS’ stands for a dilution of 10×, while 1× PBS stands for the standard solution concentration. Fluorescein isothiocyanate isomer I (FITC) (Sigma-Aldrich Co., St. Louis, MO, USA) with a concentration of 50 nM was dissolved in the solutions for visualization. To demonstrate the controlled chemical reaction using our device, the binding reaction between Fluo-4 and calcium chloride was performed. Fluo-4 (Invitrogen, Carlsbad, CA, USA) solution was prepared by dissolving the Fluo-4 powder in DI water to obtain a final concentration of 10.8 μM, while calcium chloride solution was prepared with a concentration of 5 mM.

The square waves were generated by a direct current (DC) power supply (HP Hewlett Packard 6653A, Palo Alto, CA, USA) which supplied an output voltage of 0 to 35 V, with the duty cycle controlled by LabVIEW (version 8.2, National Instruments, Austin, TX, USA). The dynamic process of the fluidic flow was monitored using an inverted optical microscope (Olympus IX71, Tokyo, Japan), and the motion was recorded by a charge-coupled device (CCD) camera (Olympus DP73, Tokyo, Japan). The exposure time was fixed at 200 ms, the magnification was set at × 6.4, and the acquired image size was 2,400 × 1,800 pixels.

The intensity of the fluorescent light was used to determine the flow rate of the proposed picoinjector. The measured fluorescent light intensity is proportional to the concentration of the fluorescent dye (FITC), and the light intensity can be analyzed from the recorded image using the image processing module in MATLAB (version 2008b, MathWorks, Natick, MA, USA). In other words, the electroosmotic flow rate can be calculated by monitoring the dynamic flowing process of the fluorescent dye from one microchannel to another via the nanochannel array. Assuming that the concentration of the fluorescent dye is *c*, the corresponding light intensity is *I*, the channel width and depth are *w* and *d*, respectively, the relation of *I* and *c* can be written as

(4) I=k×c+b

The microchannel was measured to be 1,800 × 333 (599,400 pixels) via the image capturing software, and it corresponds to the total volume of one main channel in the viewing field: *V*_*A*_ = *L* × *w* × *d* = 1,638 × 300 × 12 μm^3^ = 5,896,800 μm^3^. This means that 1 pixel in the figure stands for 5,896,800 μm^3^ / 599,400 = 9.837838 μm^3^. For another channel with the same depth of 12 μm, the concentration for each pixel is calculated by *c*_*i*_ = (*I*_*i*_ - *b*) / *k*. Thus, the corresponding volume pumped from channel A to channel B in *t*'s can be obtained from

(5) $$V=9.837838\cdot \underset{i}{\sum }\left(c_{i}/50\right)\;\left({\mu m}^{3}\right)$$

where 50 is the original concentration of FITC (50 nM) and (*c*_*i*_ / 50) is the dilution factor after pumping from one channel to another channel. Hence, the pumping rate can be calculated by

(6) $$v=\left[9.837838\cdot \underset{i}{\sum }\left(c_{i}/50\right)\right]/t$$

## Results and discussion

### Calibration of fluorescent intensity as a function of dye concentration

**Figure 3:**
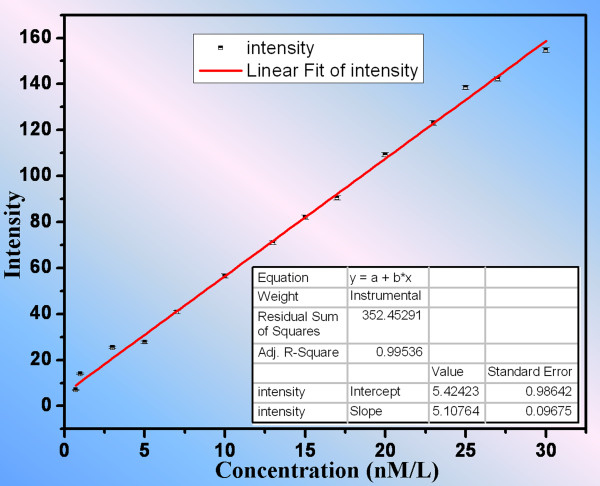
**Relation of fluorescent intensity with respect to FITC concentration in the main channel of our device.** A linear relation was obtained by fitting the data using Origin.

In order to enhance the visualization of microflow, light-emitting molecules such as fluorescent or phosphorescent ones are typically employed to increase the signal contrast [20]. In order to obtain the linear relationship of the fluorescent intensity of FITC to the dye concentration, images of microchannel filling with solutions of different dye concentrations from 0.3 to 30 nM were taken and analyzed. Fourteen sets of data corresponding to different dye concentrations were taken, and each set was measured for three times. The photo-bleaching effect was not observed in our experiment. Fluorescent intensity was analyzed by MATLAB (MathWorks, Natick, MA, USA) for each dataset. The results were plotted in Figure 3 and fitted to obtain the relation *I* = 5.1076 × *c* + 5.4242.

Here, the unit of dye concentration is nanomolar. It is noted that the interception of the fitted line is not ideally zero due to the systematic error from the CCD in detecting a very weak light signal as shown by the fluctuation in the measured intensity in Figure 3 when the dye concentration is very low (lower than 5 nM). However, the fluorescent intensity of the dye concentration greater than 5 nM indicates a good linear relation.

### Pumping rate vs. applied electric voltage

**Figure 4:**
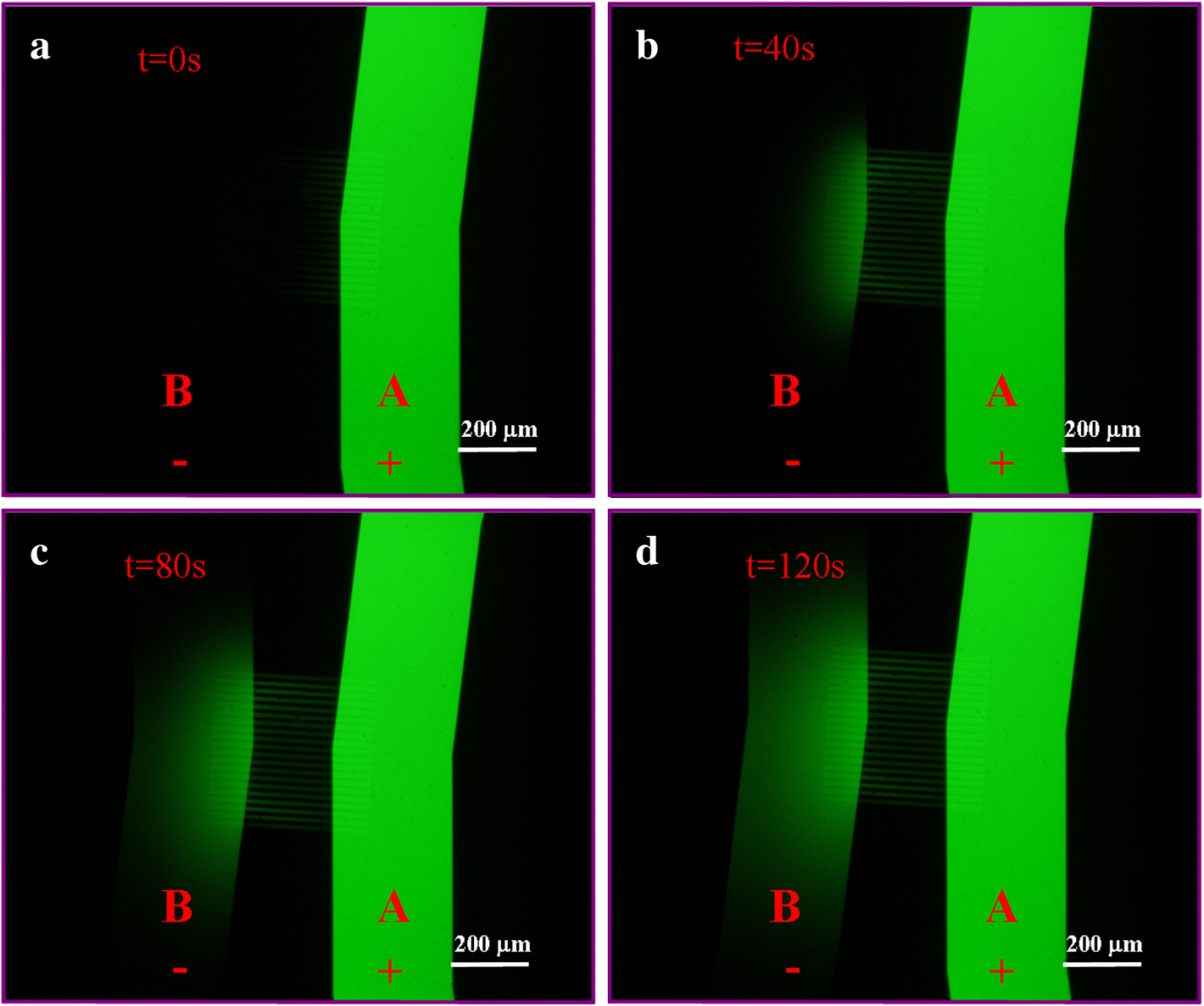
**Optical images (a-d) of the process of electroosmotic pumping from channel A to channel B.** An electric potential of 3 V was applied. Channel A contained an electrolyte solution made from 50 nM FITC dissolved in 0.05× PBS while channel B contained 0.05× PBS only. The time interval between two successive images was 40 s.

Fluid was pumped from channel A to channel B through the nanochannel array when the DC bias existed between them. It is suggested that the resulting EO flow has the same direction as the electric field for our device although the PDMS was used as one side of the square channel wall. In fact, the zeta potential of PDMS and the silica have the same polarity (both of them are negative) [21]. We measured the electroosmotic flow through the nanochannel array under the applied electric voltage in the range of 0 to 3 V with a step of 0.5 V. A time series of the flow process was recorded for determination of the flow rate. Figure 4 shows a typical dynamic process of the pumping effect with respect to the time when an electric potential of 3 V was applied. At the initial stage (Figure 4a), channel A appeared bright green while channel B was dark since channel A was filled with 50 nM FITC in 0.05× PBS and channel B was filled with 0.05× PBS. As the time elapsed, the fluid containing FITC was gradually pumped from channel A to channel B via the nanochannel array which was evident by the increase in the fluorescent intensity in Figure 4b,c,d. The diffusion of FITC from channel A to channel B was very weak compared to the effect of electroosmotic flow. No obvious fluorescent light was detected with the same acquisition setting when no electric field was applied.

**Figure 5:**
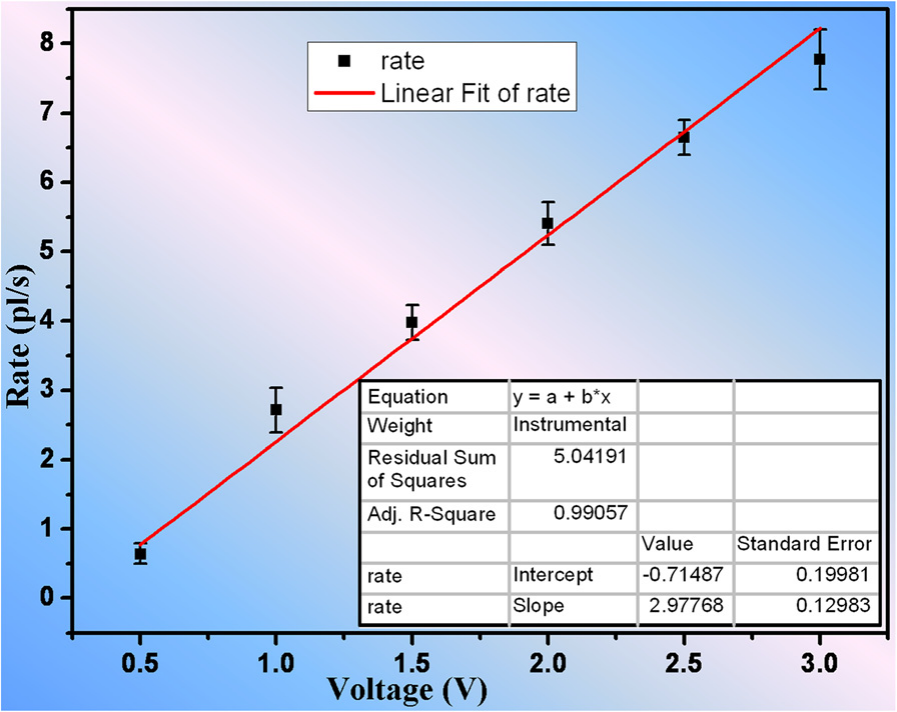
**Relation of EOF rate to the applied voltage when the electrolyte solution was 0.05× PBS.** A linear relation was obtained by fitting these data using Origin.

The averaged velocity for EO flow through the nanochannel array was determined from the temporal evolution of the pumping effect of FITC from channel A to channel B. Images were taken at every 10 s. Using Equation 6, the EO flow rates for different applied electric field values were calculated and the plot shown in Figure 5. The EO flow rate increased with the increasing electric voltage. The results were in agreement with our prediction using Equation 1 that the EO velocity is linearly proportional to the electric field strength. This relation is simply shown as *v*_EO_ = 2.9776 × *V*_EO_ - 0.7148 by linear-fitting these data in Origin. Figure 5 suggests that the precision of pumping rate can be very high (in the order of 0.1 pl/s) under the varying electric voltage. In other words, the results have implied that electric voltage could be used as a convenient means to control fluid transport with high precision, and the fabricated picoinjector has a promising potential in delivering precise control of minute amount of fluid for biochemical reactions and drug delivery systems. It is important to note that the EO mobility slightly varies at different electric field strengths [22], leading to a slight deviation especially when field strength is high, which in turns explains the fact that the interception of the line in Figure 5 was slightly smaller than the ideal number (zero).

### Effect of ion concentration

**Figure 6:**
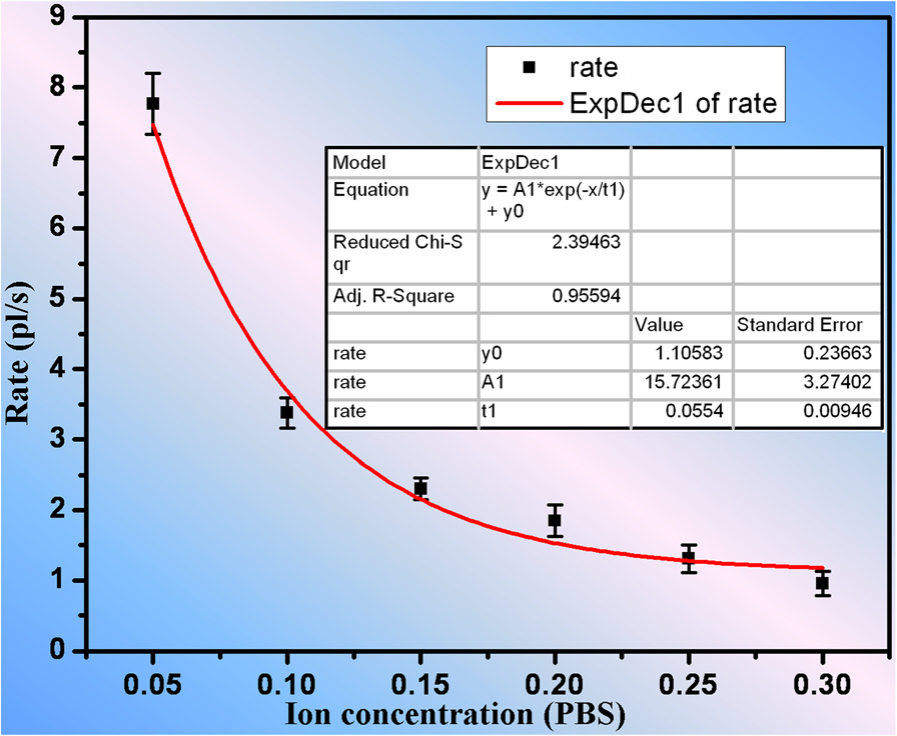
**The influence of ion concentration on the electroosmotic flow rate that exhibited an exponential function.** The ion concentration was normalized by standard PBS with a K_2_HPO_4_ concentration of 27.5 mM and a KH_2_PO_4_ concentration of 20.0 mM.

One often has to consider the practical need of transporting solutions with different analytes and concentrations. As a result, it is desirable to investigate the pumping effect of the solution with different concentrations. As reported by Tavares and McGuffin [23], the zeta potential varied linearly with the logarithm of the ion concentration, meaning that the zeta potential decayed exponentially with respect to the ion concentration. Thus, the relation between the EO flow rate and the ion concentration is an exponentially decay function under the influence of the electric field strength according to Equation 1. To examine the effect of the concentration dependency with respect to our device, the EO flow rates were measured at different ion concentrations when a constant voltage of 3 V was applied, where the ion concentration refers to the concentration of analytes in PBS in this case. The ion concentrations were normalized by the standard PBS with a K_2_HPO_4_ concentration of 27.5 mM and a KH_2_PO_4_ concentration of 20.0 mM. After analyzing the fluorescent intensity of the acquired images using imaging software, the relation of EO flow rates versus different analyte concentrations was determined and is shown in Figure 6. The analytical relation between the EO flow rate and the ion concentration was determined and exhibited exponential decay characteristics. The resulting relation is *v* = 1.10583 + 15.7236 × *e*^- 18.0505 ⋅ *c*^, where *v* is the flow rate in the unit of picoliter per second and *c* represents the analyte concentration after normalization by standard PBS. For a constant applied voltage, the higher the concentration, the lower the EO flow rate due to the decrease in zeta potential. After obtaining this relation, it is possible to estimate the flow rate of any diluted PBS driven by an applied voltage of 3 V. This method of investigating the effect of ion concentration on the EO flow rate is also applicable to other types of solution containing different analytes.

### Program-controlled reaction in continuous flow

**Figure 7:**
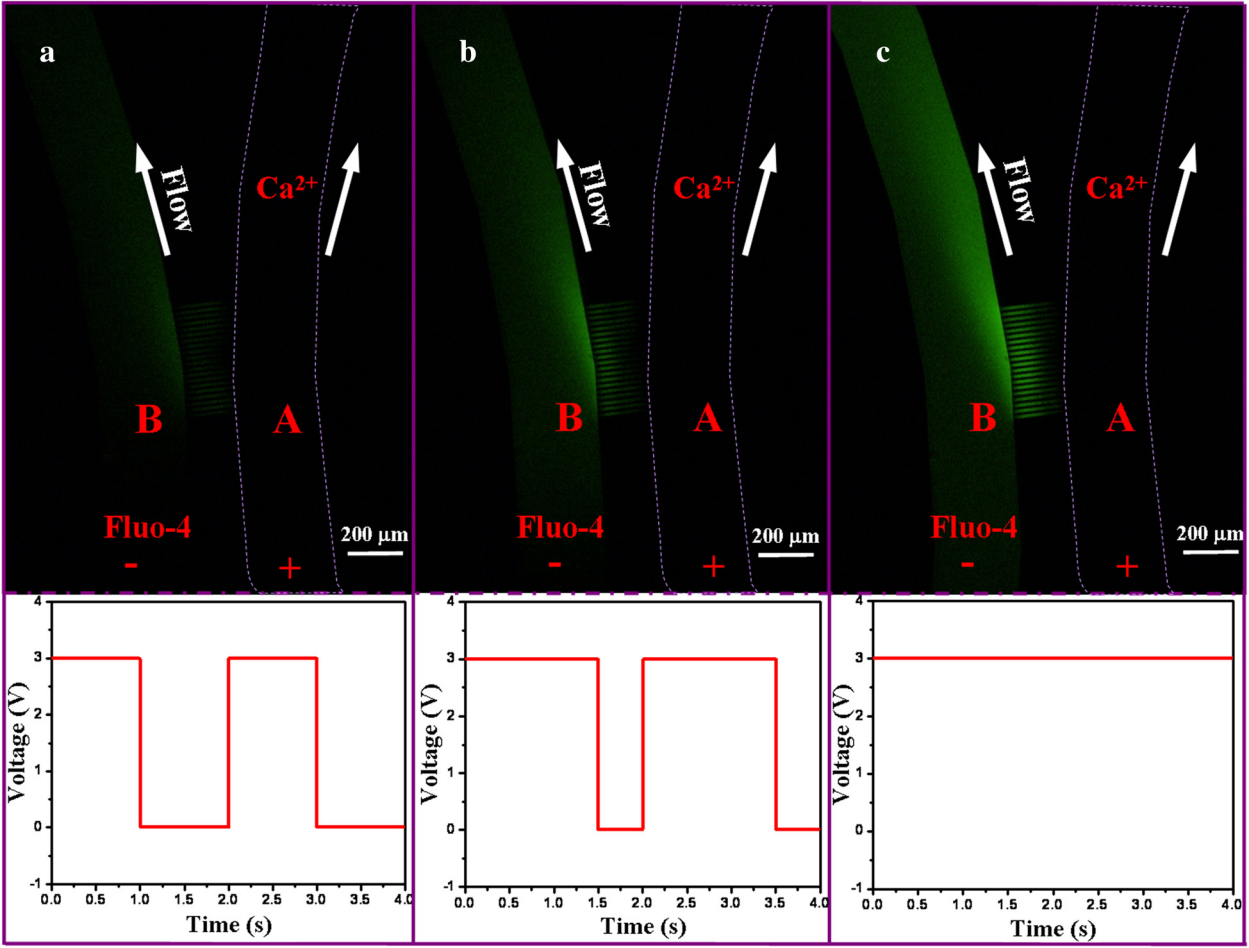
**Still optical images capturing the reaction between Fluo-4 (in channel B) and Ca**^**2+ **^**(in channel A).** The reaction is in a continuous phase and controlled by the square wave with different duty cycles: **(a)** 50%, **(b)** 75%, **(c)** 100%.

Controlled chemical reaction is one of the potential applications of our nanofluidic device, and we employed the binding reaction between Fluo-4 and calcium chloride to demonstrate the feasibility of such application. Fluo-4 is a kind of chemical widely used in living cells as a calcium indicator. Its emitted fluorescent intensity was found to be linearly proportional to the calcium concentration for a particular range [24]. Here, pumping of calcium ions was controlled by LabVIEW which generates square waves with a fixed applied voltage of 3 V and different duty cycles. The EO flow rate of the calcium chloride from channel A to channel B was measured to be 1.248 pl/s using the previously described method in determining the EO flow rate of PBS. As shown in Figure 7, Fluo-4 with a concentration of 10.8 μM flowed in channel B in a continuous phase with an apparent velocity of 40 μm/s, while calcium chloride with a concentration of 5 mM was filled in channel A. As soon as the voltage was applied across the nanochannel array, Fluo-4 bonded with the calcium ions resulting in an enhanced fluorescent intensity. The feeding quantity of the calcium ion was controlled by the effective percentage of the applied voltage with a duty cycle varying from 50% to 100%. In other words, the larger the duty cycles, the brighter (fluorescent intensity) the fluid in channel B, as indicated by comparing Figure 7a to Figure 7c. All optical images taken were at equilibrium state.

Calcium ion (Ca^2+^) is an important intracellular information transfer substance. Intracellular regulation of calcium is an important second messenger, which is widely involved in cell motility, secretion, metabolism, and differentiation of a variety of cellular functions. An accurate control of the extracellular calcium concentration is significant in many biological studies. Therefore, a real-time system with dynamic control of the calcium concentration is of great significance. We herein demonstrated the capability of our nanofluidic device for precise control of calcium concentration for biological systems.

## Conclusions

We have demonstrated that a simple nanofluidic device fabricated on a Si wafer with a thin layer of SiO_2_ and then sealed by a PDMS thin film has its potential for constructing a picoinjector. The bonding between the Si wafer and PDMS relies on the adhesion force other than chemical bonding. Therefore, it is easy to separate them, and the silicon chip could be cleaned to use repeatedly. The injection process is based on the electroosmotic flow generated by the voltage bias across the nanochannels. The EO pumping rate was measured by analyzing the fluorescent intensity when the fluorescent probe (FITC) was used in PBS as an indicator. The variations in EO flow rate at different DC voltages and different analyte concentrations were investigated, and the results exhibited good agreement with the existing theory. The precisely controlled reaction between Fluo-4 and calcium ions was used to demonstrate our device's potential application in electrochemical reaction, biochemical reaction, DNA/protein analysis, drug delivery, and drug screening. The electroosmotic effect dominates the fluid transport in our picoinjector, and electroosmosis allows our device to attain precision in fluid transport for chemical reaction on a nanoscopic scale using low DC bias voltage. The advantages of our device are its being simple, reusable, and low-cost with high precision of delivering water-based solutions.

## Competing interests

The authors declare that they have no competing interests.

## Authors' contributions

SL, WC, and YSH conducted the experiments. SL provided the physics interpretation. WW contributed most of the ideas and supervised all experiments and theory. SL, YSH, and WW wrote the paper. All authors discussed the results and commented on the manuscript. All authors read and approved the final manuscript.
